# Impact of *Toxoplasma gondii* and Human Microbiome on Suicidal Behavior: A Systematic Review

**DOI:** 10.3390/jcm13020593

**Published:** 2024-01-19

**Authors:** Ani Zerekidze, Meng Li, Alexander Refisch, Justina Shameya, Thomas Sobanski, Martin Walter, Gerd Wagner

**Affiliations:** 1 Department of Psychiatry and Psychotherapy, Jena Center for Mental Health, Jena University Hospital, 07743 Jena, Germany; 2 Circuits Underlying Mental Health (C-I-R-C), Jena-Magdeburg-Halle, 07743 Jena, Germany; 3 Department of Psychiatry, Psychotherapy and Psychosomatic Medicine, Center for Mental Health, Thueringen-Kliniken “Georgius Agricola”, 07318 Saalfeld, Germany; tsobanski@thueringen-kliniken.de; 4 German Center for Mental Health (DZPG), Partner Site Jena, 07743 Jena, Germany

**Keywords:** *Toxoplasmosis*, microbiome, microbiota, suicide, suicidal behavior

## Abstract

Background: Suicide remains a persistent global health challenge, resisting widespread prevention efforts. According to previous findings, toxoplasmosis is particularly associated with altered decision making, which could lead to risk-taking behavior, thereby increasing the likelihood for suicidal behavior (SB). In addition, discussion about the role of microbiome in psychiatric disorders has emerged lately, which also makes it relevant to investigate its role in the context of SB. Therefore, two systematic reviews are integrated in this paper, and the existing knowledge is comprehensively summarized regarding the association between microbial pathogens and SB. Methods: We conducted a systematic search with keywords including SB and *Toxoplasma gondii* (Suicid* AND Toxoplasm*) and microbiome (Suicid* AND Microbiome AND Microbiota) throughout PubMed and Scopus to retrieve related studies up to 9 November 2023, identifying 24 eligible records. The subjects of the included studies had to have fulfilled the criteria of an SB disorder as defined by DSM-5, and death cases needed to have been defined as suicide. Results: Most studies reported significant association between toxoplasmosis and SB, suggesting a higher likelihood of SB in the infected population. Regarding the microbiome, only very few studies investigated an association between SB and alterations in the microbiome. Based on six included studies, there were some indications of a link between changes in the microbiome and SB. Conclusion: The cognitive aspects of decision making in *T. gondii*-infected individuals with SB should be further investigated to unravel the underlying mechanisms. Further sufficiently powered studies are needed to establish a link between SB and alterations in the microbiome.

## 1. Introduction

Suicide remains one of the major public health concerns. Every year, more than 700,000 people commit suicide worldwide, and up to 20 times as many people attempt suicide [1]. In fact, among individuals aged 15–29 years, suicide was the third leading cause of death among girls and young women and the fourth most common cause of death among boys and young men in 2019 [2]. According to the United Nations, more people die by suicide every year than by both homicide and war [3]. Although research in the last three decades has improved our knowledge about suicide [4], it still has a limited impact on suicide prediction and prevention. Moreover, the crude suicide rate, for example, in the US rose from 11.03 to 16.14 (per 100,000), thus by nearly 46%, between 2000 and 2019 [2].

One of the problems in researching suicidal phenomena is the inconsistency of the terms previously used to describe suicidal behavior (SB) [5], leading to a variety of reported causes and risk factors [6], which has made our understanding of SB more difficult. According to the definitions provided by the United States Centers for Disease Control and Prevention, suicidal ideation (SI) corresponds to thoughts, plans, and considerations of suicide. A suicide attempt (SA) is considered a form of self-directed violence with the intention to die, often resulting in nonfatal injuries [7,8]. In 2013, the Diagnostic and Statistical Manual of Mental Disorders 5 (DSM-5) suggested “suicidal behavior disorder” (SBD) as a possible independent category [9]. SBD has been defined as a condition for further study; thus, it is not considered solely as a symptom of a major depressive disorder (MDD), bipolar disorder (BD), or borderline personality disorder (BPD). Recent studies have provided clear evidence that SB is ubiquitous in psychiatric disorders, but can also occur in a crisis without fulfilling the criteria of a psychiatric diagnosis, indicating the need to identify specific risk factors for SB and to develop suicide-specific interventions [10,11,12,13,14]. Furthermore, by explicitly differentiating SBD from nonsuicidal self-injury (NSSI), DSM-5 has put a strong emphasis on the intention of the individual at the time when the behaviors occur. Because of the complexity and multifactorial pathophysiological mechanisms leading to SB, our current knowledge to predict which persons will turn their suicidal thoughts into actions is still very limited [15].

A widely accepted explanation for the etiology of SB is the stress–diathesis model, which explains the manifestation of SB through the interaction of two main components: stress and diathesis [16]. On the one hand, acute or chronic stress factors play a role in the transition from suicidal ideations to an act: for example, interpersonal problems as well as mental disorders. On the other hand, diathesis makes individuals vulnerable to suicidal behavior. Diathesis has been operationalized as suicide-related traits that increase the chance that a person experiencing stress might respond to it through suicidal behavior [17]. For example, specific genetic, epigenetic, and neurobiological factors, such as specific polygenetic risk factors [18], specific transcriptomic features [19], decreased cortical thickness in prefrontal regions [20], or altered connectomes in individuals with SB and relatives of suicide victims [21] may contribute to this effect. Furthermore, specific psychological factors such as sensitivity to social stressors [22], abnormal processing of negative stimuli [23], and elevated levels of mental pain [24] are assumed to increase the likelihood of suicidal behavior.

In addition, alterations in neurocognitive functioning and especially decision making (DM) have been consistently found in individuals with SB [25,26], making them a characteristic feature of SB. A recent systematic review of 46 studies [27] clearly showed altered decision making in suicide attempters under conditions of uncertainty, and that they made riskier and less advantageous decisions than psychiatric and healthy controls. The authors concluded that “DM impairment could be considered as a cognitive trait of suicidal vulnerability, a risk factor and an attribute of SAs”.

In a recent publication by our group, Lubbert and [28] reported significantly higher levels of a personality trait, i.e., sensation seeking measured by a German version of the UPPS Impulsive Behavior Scale [29], in suicide attempters who used violent suicidal means, such as hanging, compared to suicide attempters using non-violent methods, such as drug overdose. Higher sensation-seeking scores may represent a clinical correlate of riskier DM in violent attempters than in nonviolent attempters, as outlined in a recent meta-analysis [30].

Furthermore, previous studies indicated a potential association between neurocognitive deficits and inflammatory biomarkers [9,31,32]. As reported in the review by Courtet and Giner [33], a relatively consistent association between suicide and inflammatory cytokines in the orbitofrontal cortex, the brain region involved in the process of reward-related decision making and affective control, was demonstrated in post mortem studies. Possible involvement of microglial cells, which are activated during neuroinflammation, has also been discussed in connection with suicidal behavior [34]. Significantly higher levels of peripheral C-reactive protein (CRP) have been found in individuals with suicidal behavior in contrast to participants with depressive disorders as well as healthy individuals [35]. However, there is a limited understanding of potentially relevant factors such as the gut microbiome or toxoplasmosis, which may act as internal and external triggers for SB. Even if previous exposure and seropositivity to pathogens, such as herpes, Epstein–Barr virus (EBV), cytomegalovirus, etc., have been implicated in multiple psychiatric conditions [36], there are several plausible reasons to believe that changes in the human microbiome and toxoplasmosis may have a specific link to suicidal behavior.

For example, the risk of suicide is 17 times higher in people with mood disorders than in the general population, and follow-up studies have documented that ten to fifteen percent of patients with major depressive disorder (MDD) die by suicide during the course of the disease [37]. Previous studies have suggested that alterations in the gut microbiome are associated with metabolic disturbances and inflammatory processes in individuals with mood disorders [38]. Since the gut microbiome has a strong influence on immune-inflammatory processes [39,40] and, in particular, on the stress response [41], both of which have also been shown to be altered in people with SB [17], changes in the gut microbiome could, thus, further increase the risk of suicidal behavior.

In addition, it has been shown that animals and humans infected with *Toxoplasma gondii* often make risky decisions, which is a characteristic feature of suicidal behavior. Moreover, toxoplasmosis can also lead to inflammatory processes in the central nervous system (CNS), affecting the host’s behavior. There is some evidence of a link between toxoplasmosis and suicidal behavior, which will be presented in detail in this systematic review.

Therefore, in this paper, we have focused specifically on the association between the human microbiome, toxoplasmosis, and suicidal behavior, for which we will present the available evidence.

### 1.1. Toxoplasmosis and Suicidal Behavior

Toxoplasmosis is a common zoonotic disease caused by the intracellular protozoan *Toxoplasma gondii* (*T. gondii*), which exists in roughly one-third of the world’s population [42]. The global prevalence of toxoplasmosis is continuously rising. According to the U.S. Centers for Disease Control and Prevention, over 60 million individuals in the United States may have contracted the infection, and in certain regions, prevalence rates are as high as 95% [43]. The most common routes of disease transmission to humans are through oral ingestion of tissue cysts containing bradyzoites, often found in undercooked or raw meat, in food containing oocysts excreted in the feces of infected cats, or through the consumption of contaminated water [44,45]. On rare occasions, infection can be obtained through the transfer of tachyzoites from a pregnant mother to her unborn child across the placenta or via blood transfusions and organ transplants [46]. The clinical representation of infection in healthy subjects is, in about 90% of cases, asymptomatic or only with mild symptoms. However, in pregnancy, the parasite might lead to several complications, such as miscarriage and damage to the baby’s eyes and brain, which later lead to psychomotor or mental disorders in infants [47].

According to earlier findings, toxoplasmosis was particularly associated with altered decision making in nonhumans and humans. Animal models have demonstrated a strong relationship between *T. gondii* infection and increased risk-taking behaviors [48]. For example, mice infected with *T. gondii* appear to exhibit altered behavior, i.e., they are attracted to cats instead of avoiding them, which increases the likelihood that they will be caught. They are more likely to explore the novel areas in mazes and display themselves in open spaces [49,50]. Risk-taking behavior has been observed not only in *T. gondii*-infected rodents, but also in nonfeline, chronically infected mammals, such as wolves and chimpanzees. Wolves with a positive serological status demonstrated a higher tendency to take high-risk actions such as dispersal and assuming the role of a pack leader [51]. Furthermore, Toxoplasma-infected chimpanzees showed a loss of innate aversion toward the urine of their natural predators. They more frequently approached and investigated leopard urine than noninfected individuals [52]. The changes in the brain associated with the infection that leads to increased risk behavior are complicated and not fully understood. However, a high parasite cyst density has been shown in the amygdala [50], an important brain region for the processing of fear and, more generally, for the recognition of salient information.

Similar changes in decision making have been suspected in *T. gondii*-infected humans. Similar to infected mice, humans might also be more likely to show risky behavior when carrying latent toxoplasmosis, for instance, not wearing a helmet while riding a motorcycle, engaging in traffic accidents, or attempting suicide [53,54,55]. Retrospective examinations have shown significant associations between *T. gondii* seropositivity and outcomes such as car accidents, drug abuse, and suicide [56]. These results illustrate that parasites might carry significant consequences for intermediary hosts, extending beyond immediate infections to influence behavior. In social species, these effects can extend beyond individual organisms, even impacting groups through altering social interaction patterns. Therefore, we hypothesize that toxoplasmosis plays a role in SB by altering decision-making processes toward riskier choices.

Several studies have examined *T. gondii* seropositivity among suicide victims and suicide attempters. However, the previous findings are inconsistent, and the underlying mechanisms remain unclear. In the most recent meta-analysis, Soleymani and Faizi [55] systematically reviewed sixteen studies in which eight papers reported no association, seven papers revealed a positive association, and one revealed a negative association between *T. gondii* and suicide. Nevertheless, the overall rate of suicide was higher in people with *T. gondii* infection than in those without infection.

Postolache and Wadhawan [57] have systematically reviewed the three previous meta-analyses [55,56,58] reporting an association between *T. gondii* and suicidal behavior. The results were relatively consistent in effect size in all three reviews, showing a higher overall rate of suicide attempts in people with *T. gondii* infection than in those without infection.

Taking into account that Soleymani and Faizi [55] only included studies published until March 2019 and that the definition of suicidal behavior in previous studies was often unclear, we have conducted a systematic search of the papers reporting a possible link between *T. gondii* and SB to test our hypothesis. To reduce potential heterogeneity between previous studies due to the different definitions along the spectrum of suicidal phenomena, studies which made a clear distinction between suicidal behavior and suicidal ideation or NSSI in relation to the intent to die, according to DSM-5, were included in this systematic review.

### 1.2. Gut–Brain Axis

The gut–brain axis (GBA), i.e., the interaction of the gastrointestinal tract and the brain, has become a recent focus of psychiatric research and is increasingly recognized as an emerging area that could promote a better understanding of mental disorders and help to develop new diagnostic and therapeutic biomarkers [59]. The GBA is a bidirectional and dynamic communication network between the gut and brain that is modulated by the gut microbiota, which refers to the collection of all microorganisms living in the gut. Dysbiosis, i.e., changes or imbalance in the microbial composition [60], results in changes in the production of microbial metabolites, e.g., including short-chain fatty acids (SCFAs) like acetic acid, propionic acid, and butyric acid, which have significant functions in the central nervous system [61]. The gut microbiome has a significant impact on the development of the hypothalamic–pituitary–adrenal (HPA) axis, which is primarily known for its role in mediating the stress response. Sudo and Chida [62] demonstrated that germ-free (GF) mice, compared to control mice, responded to mild stress with an increased release of adrenocorticotropic hormone (ACTH) and corticosterone. Conversely, chronic stress could be associated with long-lasting effects on various aspects of immune homeostasis, reduced diversity in the microbiome, increased susceptibility to infectious diseases, and altered inflammatory responses, as has been observed in various psychiatric disorders [39].

In recent decades, accumulating research has shown that the interplay between the brain, gut, and microbiota is implicated in the development of depression [38,63,64,65]. The vast majority of our understanding of how microbes affect mental function has come from preclinical studies in rodent models [66]. However, a growing body of research analyzing the microbiota in clinical cohorts has emerged, revealing variances across diverse taxonomic levels, albeit with occasional inconsistencies in the findings. Most consistently, elevated levels of bacteria have been linked to increased inflammation and a decrease in anti-inflammatory bacteria. Furthermore, the microbiome is intricately connected to numerous physiological aspects relevant to depression, encompassing neurotransmission, neural plasticity, stress modulation, immune response, and metabolic factors [39]. Environmental factors such as smoking, alcohol consumption, and diet could have a significant influence on the composition of the microbiota in individual organisms [67]. Previous studies have suggested, therefore, that alterations in the gut microbiota are also associated with metabolic disturbances in individuals with depression [68], which might be explained by differences in diet. A longitudinal study revealed a correlation between poor nutrition and depression, whereas a healthy diet was associated with lower depressive symptoms [69]. Not only in individuals with an affective disorder, but also in individuals with SB, an association between fast food consumption and past SB was reported when controlling for food insecurity, obesity, physical exercise, and consumption of fruits and vegetables in 32 countries [70]. People at risk of suicide appear to exhibit negative health-promoting behavior [71], which might be associated with alterations in the microbiome. Very few studies have investigated the association between suicidal behavior and the microbiome. To date, no systematic review has been conducted summarizing the existing knowledge about the association between the microbiome and suicidal behavior. We aimed to outline the role of the microbiome as an additional internal factor that might increase vulnerability to suicidal behavior, as well as to summarize actual findings.

The aim of this systematic review is, therefore, to summarize and to evaluate the findings on the role of *T. gondii* and of the human microbiome in suicidal behavior and to discuss possible mechanisms of how they could contribute to the transition from SI to SB.

We deliberately did not limit the search to the gut microbiota alone, as this is the first review on this topic. We also aimed to comprehensively investigate potential changes in the microbiome across different anatomical regions.

## 2. Methods

### Search Strategy

We adopted the systematic review guidelines provided by the Transparent Reporting of Systematic Reviews and Meta-Analyses (PRISMA) statement (see Supplementary Materials) [72]. We conducted a systematic search throughout the National Center for Biotechnology database on PubMed (PMC) to retrieve related studies up to 9 November 2023. Two major keywords, suicide and *T. gondii* (Suicid* AND Toxoplasm*), were used to construct a search strategy for database. With regard to microbiome, we used the keywords microbiome, microbiota, and suicide (Microbiome OR Microbiota AND Suicid*), which that were entered into the advanced search of the PMC database with the filter (Body—All Words). We also performed an additional search on Scopus using specified terms sought through abstracts (ABS) (ABS (microbiome) OR ABS (microbiota) AND ABS (suicid*)) and (ABS (toxoplasm*) AND ABS (suicid*)) to ensure that we did not overlook any additional relevant publications. In addition to our search strategy, reference lists of previous relevant systematic reviews and cross-references via Google Scholar were examined to identify further eligible studies that may have been missed by the search algorithm used.

According to the Population, Intervention, Comparison and Outcomes (PICOs) principles, we systematically selected studies, including case–control, cohort, and cross–sectional studies, that reported an association between *T. gondii* infection or the microbiome or microbiota (as a predictor) and suicide (as an outcome) in all age and sex groups of the population, including post mortem studies. We selected studies assessing the association of *T. gondii* or microbiome with suicidal behavior based on the following inclusion criteria:

1. Studies, including case–control, cohort, and cross–sectional, both pre mortem and post mortem;
2. Studies published in English;
3. Studies published up to 9 November 2023, with no further time limitation;
4. Studies investigating a potential association between *T. gondii* infection or microbiome or microbiota and suicidal behavior;
5. Studies including individuals with suicidal behavior meeting the criteria of SBD as defined by DSM-5;
6. Participants with self-harm were included only if the intent to die or an expectation of the lethality of a suicide attempt was identifiable in the study’s definition of self-harm;
7. Studies that assessed suicide attempts using standardized methods such as the Columbia Suicide Severity Rating Scale (C-SSRS);
8. Post mortem studies including death cases defined as suicide.

Studies were excluded if they met any of the following exclusion criteria:

1. Systematic reviews or meta-analyses;
2. Published in a language other than English;
3. Studies that did not explore a potential association between *T. gondii* infection or microbiome or microbiota and suicidal behavior;
4. Studies exclusively involving individuals with suicidal ideations;
5. Studies with unclear definitions of suicidal behavior, or those assessing suicidal behavior with self-rating questionnaires or unstandardized assessment tools;
6. Studies including individuals engaging in self-harm without the intent to die.

Two independent reviewers (A.Z. and J.S.) searched the databases and screened the titles, abstracts, and full texts of the studies to choose the relevant studies.

## 3. Results

### 3.1. Toxoplasmosis and Suicidal Behavior

**Figure 1:**
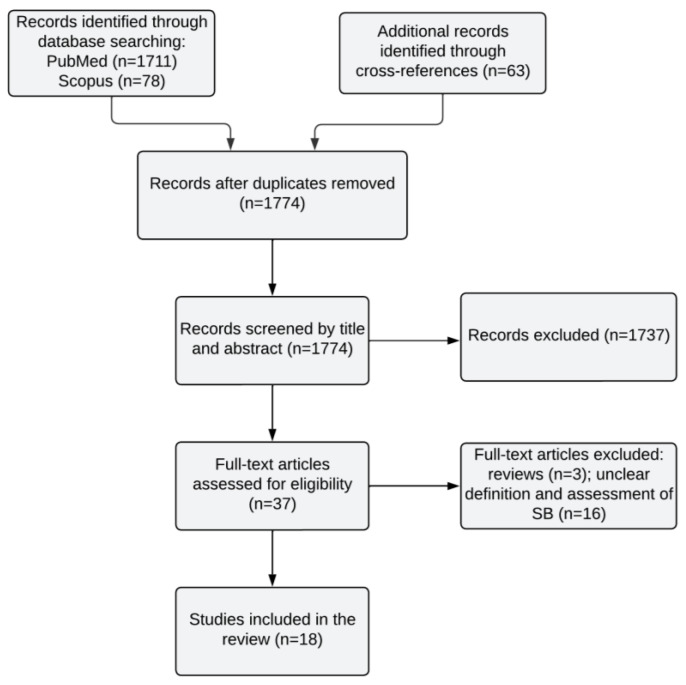
Flowchart of the selection process for the studies investigating a link between *Toxoplasma gondii* and suicidal behavior.

The systematic search of the databases yielded 1852 studies, eighteen of which were included in the systematic review after removing the duplicates and assessed for eligibility (see Figure 1). Three reviews [55,56,57] and sixteen studies [73,74,75,76,77,78,79,80,81,82,83,84,85,86,87,88] with unclear definitions of or assessment tools for SB were excluded. The characteristics of these studies are displayed in Table 1.

Overall, twelve out of eighteen studies reported a statistically significant positive relationship between *T. gondii* and suicidal behavior [46,89,91,92,93,94,95,96,97,98,99,103], and one study reported a significant relationship on the trend level [100], while five studies did not find any such association [90,101,102,104,105]. However, Samojlowicz and Borowska-Solonynko [101] did find a statistically significant result with respect to the seroprevalence of *T. gondii* in the 38–58 age group when analyzing the subgroups. Thus, most studies demonstrated an association between *T. gondii* seropositivity and suicidal behavior. However, one study additionally reported a significant association between suicide attempts and positive blood alcohol tests [103], which could be considered a confounding factor. Five studies investigated the relationship between serointensity and suicidal behavior. The results were contradictory: Three out of the five studies reported positive associations with SA in terms of higher odds ratios [94,96,98], while in the other two studies, the association was nonsignificant [90,91].

In three post mortem studies [90,101,103], anti-*T. gondii* IgG antibodies were measured in blood samples. These studies did not reveal an overall association between *T. gondii* seropositivity and suicide. However, when examining subgroups, a statistically significantly higher frequency of *T. gondii* seropositivity was observed in the 38–58 age group [100]. The fourth post mortem study [89] investigated *T. gondii* immunohistochemistry in brain tissues from the prefrontal cortex and amygdala. This study reported a positive immunohistochemistry, mainly in the prefrontal cortices of suicide decedents.

### 3.2. Microbiome and Suicidal Behavior

**Figure 2:**
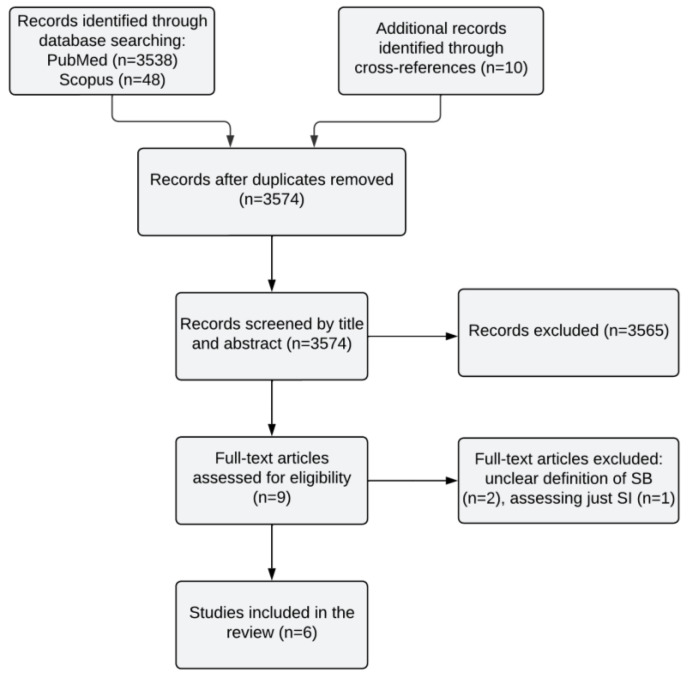
Flowchart of the selection process of studies investigating a link between microbiome and suicidal behavior.

Through a systematic search on PubMed and Scopus, we identified 3586 papers. Ten further studies were found by checking the cross-references. After screening and assessing for eligibility, three records were excluded [106,107,108] because of the unclear assessment of SB or missing information. Consequently, six papers meeting the inclusion criteria were included in this review (Figure 2).

Table 2 presents the characteristics of the six eligible studies. Five out of six studies reported a significant association of suicidal behavior with the microbiome [109,110,111,112,113]. One well-powered study investigating fecal swabs collected from 100 psychiatric inpatients did not find any significant relationship between gut microbiota composition and suicide-related behavior [114]. However, only 35 individuals out of the included 100 exhibited past suicidal behavior, which significantly dilutes the interpretation of the result. No separate analyses for the SB or SI groups were reported.

In all three post mortem studies, altered microbiome composition was found in suicide victims. Suicide was significantly associated with lower levels of Peptostreptococcaceae, [110] and higher levels of Actinomyces [113], both gram-positive bacteria. Moreover, significant differences in Chao1 richness [109] in suicide victims were reported. A study examining stool samples revealed a significant relationship between the enterotype dysbiosis index and suicidal behavior in individuals with MDD [111]. By investigating plasma zonulin, a marker of gut permeability [115], it was found that the SB group had lower zonulin levels and, additionally, higher levels of intestinal fatty acid binding protein (I-FABP) compared to depressed individuals without SB and unaffected controls [112].

## 4. Discussion

The goal of this systematic review was to comprehensively summarize the existing knowledge regarding the association between *T. gondii* and the microbiome and suicidal behavior according to DSM-5 criteria. Therefore, we conducted two systematic searches, identifying a total of 5448 records with our search algorithm, and included 24 studies in this review. Twelve out of eighteen studies found a positive association between *T. gondii* and suicidal behavior, and there were small indications of a connection between changes in the microbiome composition and suicidal behavior. The results indicate an additional role of external and internal microbial pathogens in increasing the risk of suicidal behavior.

### 4.1. Toxoplasmosis

The goal of the first review was to comprehensively summarize the existing knowledge regarding the association between *T. gondii* and suicidal behavior. We assumed that the parasite could influence the decision-making process in intermediate hosts, leading to risky behavior in *T. gondii*-infected individuals and thereby increasing the likelihood of a suicidal act. To test our hypothesis, we screened 1774 papers and incorporated 18 reports into the review. Most of the results supported the assumption of a positive correlation between *T. gondii* and suicidal behavior. This means that individuals infected with *T. gondii* appear to be more prone to suicidal behavior, making the resulting infection an additional external factor contributing to the transition from suicidal ideation to action.

The underlying mechanism in humans appears to be altered decision making in terms of lower risk aversion as a result of the infection. Relating to animals, the manipulation hypothesis says that the parasite’s manipulative activity aims to shift the intermediate host’s response from aversion to attraction, facilitating the chasing and eating of the infected host and increasing the likelihood of transmission from an intermediate to a definitive host [116]. The mechanisms behind the conversion from aversion to attraction are complex and still a topic of discussion. Based on the results of neurobiological studies, the conversion could be explained by the effects of *T. gondii* on the function of certain brain regions.

Although parasite cysts are distributed throughout the entire brain of infected rodents, the amygdala appears to be particularly affected. The density of cysts found in the amygdala has been found to be twice as high as in other regions of the brain [50], having a potential impact on fear processing or detection of other salient stimuli. This dysfunction of the amygdala and, in addition, of the prefrontal cortex [89], could change risk-taking behavior through the altered perception of the threat and, thus, of impending negative consequences. There is also some, but still little, evidence of the effects of toxoplasmosis on the structure and function of the human brain [89].

In view of the available evidence for the neuronal effects of acute toxoplasmosis, further studies are urgently needed to determine the neuronal correlates of the changes in infected populations and their relationship to altered neurocognitive processes. To date, no study has examined the clinical profiles and cognitive aspects of decision making in *T. gondii*-infected individuals with SB, which may be a promising intervention target for future psychotherapeutic and pharmacological approaches. The latter therapies should be effective against both tachyzoites and bradyzoites that remain in the body for life.

Several limitations within the included studies could lead to challenges in the interpretation of our results. Although we included studies with clearly defined criteria for suicidal behavior disorder according to the DSM-5, inconsistencies with regard to existing mental illnesses (e.g., schizophrenia, affective disorders), the composition of the control groups (healthy controls vs. patient controls), study design (e.g., cross-sectional, case–control, cohort), and biological samples (e.g., brain tissue, plasma) may have contributed to the heterogeneity of the findings.

Additionally, two studies reported the association of *T. gondii* with SB in people with significant alcohol levels in their blood. Alcohol intoxication could serve as a trigger for the transition to suicidal behavior, as alcohol itself could influence decision making, interfering with the effect of toxoplasmosis [117]. Another limitation refers to the lack of information on the socioeconomic status of the included individuals, which might also be a potential confounder. Furthermore, the prevalence of *T. gondii* is the highest in Africa [118]; however, the studies included in this review exclusively have been published in high-income countries, and therefore, one should be careful about drawing general inferences regarding developing and emerging countries.

Moreover, the inclusion of four post mortem studies, focusing on post mortem blood or brain tissues in suicide victims, adds an additional layer of potential variation. The anti-*T. gondii* IgG antibodies were measured with an enzyme-linked immunosorbent assay (ELISA) kits, which are typically designed and validated for use with fresh or frozen clinical samples. The post mortem studies did not provide further information about the validation of the method for post mortem blood samples, which could be a source of an error for the results.

In addition, reports indicate that the probability of suicide is significantly higher among individuals who use violent suicide means than among those using nonviolent methods [119,120]. Therefore, violent suicide attempters using high-lethality methods might comprise a specific subgroup with very high-risk behavior, closer to suicide victims regarding psychopathological and neurocognitive features such as decision making [121,122,123]. However, the studies included in the present systematic review did not provide any information about the methods used for SB. The lack of this suicide-specific information in these studies may also contribute to some variability in the reported findings.

### 4.2. Microbiome

The present study is the first review summarizing the existing knowledge about the association between the microbiome and suicidal behavior. We hypothesized that there might be alterations in the microbiome in suicide attempters or suicide victims; therefore, through the systematic search, we identified six studies investigating this relationship. Five studies demonstrated an association between changes in the microbiome and suicidal behavior. Only Thompson and Fowler [114] reported no significant relationship between gut microbiota composition and “suicidality” in a cohort of mentally ill people. In this study, stool specimens of a large sample of 100 people with and without suicidal thoughts and behavior were analyzed, forming a homogeneous sample in terms of medication status. However, only 35% of the investigated individuals in that study showed actual suicidal behavior, and most individuals only expressed suicidal thoughts, which considerably limits the interpretation of the results.

In contrast, the second study, analyzing stool samples, reported an association between suicidal behaviors and the dysbiosis index in the depression enterotype [111]. All post mortem studies revealed an association or a trend between the microbiome in post mortem organ tissues and suicide. However, it is crucial to recognize the potential impact of the post mortem breakdown of the gut barrier, leading to the translocation of bacteria throughout the body. Impairment of cellular integrity after death leads to the breakdown of the tight junctions between epithelial cells that normally prevent the entry of harmful microorganisms, toxins, and substances into the bloodstream [124]. The primary sterile organs, such as the liver, spleen, brain, or other organs, are affected by the microbiome post mortem. Therefore, we might hypothesize that dysbiosis in the gut might be reflected in other anatomical regions as well after death. However, the inherent variability in post mortem microbiome composition, i.e., after the first 24–48 h after host death [125], raises questions about the reliability of study results. Thus, the composition of the thanatomicrobiome is influenced by the time period between death and autopsy [126], which adds a layer of complexity to the interpretation of the results.

As the results are controversial, further investigation is needed to establish whether there is an association between suicidal behaviors and alterations in the gut microbiota. To summarize, the number of identified studies is small, and the heterogeneity of the applied methods is relatively large, so a clear conclusion about the role of the microbiome in SB is currently not possible. Medication, which is often prescribed to treat mood disorders, is an important factor to consider due to its influence on the composition of the gut microbiome [127]. Thus, the use of healthy individuals as a comparison group, as observed in two studies, represents a potential confounding factor, particularly with regard to the use of medication. Different biological samples, including brain or other organ tissues, blood, and stool samples, might also lead to differences in results. Further limitations, such as differences in the types of mental disorders of the individuals and study design, must also be taken into consideration.

Experimental clinical studies investigating the role of the gut microbiome in suicidal behavior are lacking. Most of the current research focuses on depressed people who show signs of an altered microbiota composition [39,41]. A recent comprehensive population study demonstrated that the Coprococcus and Dialister strains not only acted as predictors of an enhanced quality of life, but were also consistently found to be reduced in untreated individuals with depression [128]. As discussed above, the gut microbiome has an impact on the HPA axis, which itself affects how individuals react to stress. Interestingly, recent studies have demonstrated that fecal matter transplantation from nonstressed animals partially reduces certain harmful effects of stress [129]. Conversely, transplanting fecal matter from stressed to naïve mice was sufficient to induce stress-related effects in the recipient mice [129]. Moreover, transplanting fecal material from stressed mice to previously unaffected recipients led to anxiety-like behavior and the accumulation of monocytes and activation of microglia in the hippocampus, while administering beneficial microbes from nonstressed animals improved anxiety-like behavior by alleviating inflammation in the gut [130]. These findings indicate the involvement of the gut microbiome in the stress response. Since it is assumed that an abnormal HPA axis and stress response [131] play a decisive role in the development of suicidal behavior, changes in the microbiome due to genetic/epigenetic mechanisms or due to reduced health-related behavior could be an additional factor that increases susceptibility to suicidal behavior.

In addition, the gut microbiome has a crucial role in neuroendocrinological functions and brain development. Microbial metabolites such as short-chain fatty acids (SCFAs) regulate intestinal immune function and have anti-inflammatory properties, particularly in inhibiting proinflammatory mediators in macrophages and influencing the maturation and function of microglia [132,133]. Emerging evidence suggests that the gut microbiota can influence intestinal barrier permeability and ultimately lead to the dysregulation of inflammatory responses [134]. There are some indications of a possible link between inflammation in both the peripheral and central nervous systems and suicidal behavior [135]. Therefore, we propose that the likely mediating role of the microbiome in inflammatory and stress-related processes associated with suicidal behavior be systematically investigated as a focus of future research.

Finally, as the assessment of risk of bias in non-randomized studies is generally not feasible with tools such as the Cochrane Risk of Bias, we have provided detailed information on the included studies, e.g., assessment methods, included subjects, study design, statistical parameters, etc., as shown in Table 1 and Table 2, to enable the reader to assess the quality of the included studies.

## 5. Conclusions

In this systematic review, we have demonstrated the association between *T. gondii* and suicidal behavior according to the DSM-5 definition and discussed the probable underlying mechanisms. However, in order to better understand the mechanism of toxoplasmosis, further specific studies on the influence of the parasite on risky decision making in vulnerable individuals need to be performed. Furthermore, this is the first review summarizing the existing literature on the likely link between changes in the microbiome and suicidal behavior. There is some evidence that the microbiome is an additional internal factor that may increase vulnerability to suicidal behavior. However, future systematic and sufficiently powered studies should additionally investigate the mediating role of the microbiome in inflammatory and stress-related processes associated with suicidal behavior. Finally, in the present review, we deliberately focused on the association between Toxoplasma gondii and the human microbiome in relation to suicidal behavior, as we have a strong hypothesis that, unlike other pathogens, this is the case. However, the role of other microorganisms or viruses in suicidal behavior needs to be considered, which should be addressed in future studies.

## Figures and Tables

**Table 1:** Individual studies used in this review reporting the association between *T. gondii* and suicidal behavior.

Study	Country and Year	Design	SB Definition	Age in Years (Mean and First SD)	Number of Patients	Number of Control Participants	Gender (N)	Type of Biological Sample	Main Outcome
Alvarado-Esquivel, Mendoza-Larios [89]	Mexico, 2021	Cross-sectional	Decedents who died by suicide based on medico-legal autopsies.	34.8 ± 17.4	87 suicide victims	-	P: (M: 67, F: 20)	Post mortem brain (prefrontal cortex and amygdala)	A history of depression was associated with *T. gondii* infection of the brain in suicide victims (OR: 12.00; 95% CI: 2.26–63.46; *p* = 0.003).
Mendoza-Larios, Garcia-Dolores [90]	Mexico, 2021	Case–control	Decedents who died by suicide and were studied after forensic examination.	P: 35.21 ± 17.48 HC: 31.82 ± 15.01	89 decedents who committed suicide	58 decedents	P: (M: 68, F: 21) HC: (M: 48, F: 10)	Post mortem plasma	No association between *T. gondii* seropositivity and suicide (OR: 0.85; 95% CI: 0.28–2.60; *p* = 0.78).
Bak, Shim [91]	South Korea, 2018	Case–control	SA according to Columbia Suicide Severity Rating Scale.	P: 43.75 ± 16.75 HC: 41.59 ± 11.54	155 inpatients and outpatients with depressive symptoms + SA	135 HC	P: (M: 75, F: 80) HC: (M: 66, F: 69)	Plasma	Suicide attempters showed higher seroprevalence of *T. gondii* than healthy controls (OR: 2.49; 95% CI, 1.26–4.93; *p* = 0.011).
Coryell, Yolken [92]	USA, 2016	Case–control	SA was defined as any self-harm that was intended to cause death regardless of premeditation or potential lethality.	P: 17.5 ± 1.7 PC: 19.0 ± 1.6	17 individuals with MDD + SA	91 Individuals with MDD	P: (M: 4, F: 13) PC: (M: 26, F: 65)	Plasma	A significantly higher toxoplasmosis IgG titer among individuals who had recently begun a trial of SSRIs and had a history of suicide attempts (t = −2.67, df = 106, *p* = 0.009).
Demirel, Akgul [93]	Turkey, 2023	Case–control	Structured clinical interview for DSM-5.	MDD: 39.90 ± 13.95 BD: 35.88 ± 12.38 HC: 38.30 ± 14.20	83 individuals with MDD + SA; 93 individuals with BD + SA	78 individuals with MDD; 54 individuals with BD; 310 HC	MDD: (M: 71, F: 76) BD: (M: 73, F: 88) HC: (M: 149, F: 161)	Plasma	Stratified analyses of the binary outcome data indicated that *T. gondii* seropositivity was a predictor of individuals with a history of suicide attempts (OR = 17.17; 95% CI [8.12–36.28]; *p* < 0.001).
Coryell, Wilcox [94]	USA, 2020	Cross-sectional	SA according to Columbia Suicide Severity Rating Scale.	P: 32.6 ± 13.1 PC: 38.2 ± 15.4	96 individuals with MDD + at leasr two SA	128 individuals with MDD	P: (M: 33, F: 63) PC: (M: 37, F: 91)	Plasma	*Toxoplasma gondii* IgM levels were higher, and seropositivity more likely, in suicide attempters (X^2^ = 7.4, *p* = 0.01)
Ling, Lester [95]	WHO, Europe, 2011	Ecological Study	Suicide rates by age group were obtained from the European Mortality Database.	Range 0–75+	432,974 individuals		Only females	Plasma	Positive relationship between rates of infection with *T. gondii* and suicide is apparent in women of postmenopausal age (t = 3.02, standardized beta, 0.62, *p* = 0.007)
Zhang, Traskman-Bendz [96]	Sweden, 2012	Cross-sectional	Situations in which a person has performed an actually or seemingly life-threatening behavior with the intent of jeopardizing his/her life or to give the appearance of such intent, but which has not resulted in death.	P: 38.4 ± 14.4 HC: 39.8 ± 14.2	54 individuals with mixed diagnosis + SA	30 HC	P: (M: 23, F: 31) HC: (M: 11, F: 19)	Plasma	Seropositivity of *T. gondii* (OR = 7.12; 95% CI, 1.66–30.6; *p* = 0.008) and serointensity of *T. gondii* (OR = 2.01; 95% CI, 1.09–3.71; *p* = 0.03) were positively associated with a history of SA.
Akgul, Demirel [97]	Turkey, 2021	Case–control	SA according to Suicide Behaviors Questionnaire—Revised and clinical interviews.	P: 47.51 ± 24.83 PC: 43.96 ± 18.33 HC: 42.27 ± 29.11	57 individuals with schizophrenia + SA	60 individuals with schizophrenia 120 HC	P: (M: 34, F: 23) PC: (M: 32, F: 28) HC: (M: 53, F: 67)	Plasma	The relationship between the history of SA and seroprevalence of *T gondii* was found to be statistically significant (*p* < 0.05). The history of SA was not statistically associated (*p* = 0.831) with *T gondii* positivity by PCR.
Kamal, Kamal [46]	Egypt, 2022	Cross-sectional Case–control	Columbia Suicide Severity Rating Scale (C-SSRS)	P: 32.39 ± 10.47 HC: 33.10 ± 11.03	384 depressed individuals	400 HC	P: (M: 209, F: 175) HC: (M: 214,F: 186)	Serum	Seropositive depressed participants were more likely to have prior history of SA compared with seronegative participants (OR= 6.2, 95% CI: 3.4–11.2, *p* < 0.001).
Dickerson, Wilcox [98]	USA, 2017	Cross-sectional	A suicide attempt was defined as a potentially self-injurious act committed with at least some intent to die as a result of the act.	P: 38.6 ± 13.0 PC: 36.5 ± 13.8	72 individuals with psychiatric diagnosis + SA	90 patient controls	P: (M: 38, F: 34) PC: (M: 50, F: 40)	Plasma	Higher odds of a suicide attempt history in individuals who had elevated levels of IgM antibodies to *T. gondii* (OR = 2.41, 95% CI 1.02, 5.71, *p* = 0.046); A significant correlation between a lifetime history of suicide attempts and the level of IgM class antibodies (beta = 0.070, 95% CI 0.007, 0.134, *p* = 0.03).
Arling, Yolken [99]	USA, 2009	Case–control	The Columbia Suicide History Form, a semi-structured questionnaire, was used to assess suicide attempt history.	P: 40.3 ± 9.8 PC: 43.4 ± 10.9 HC: 42.7 ± 11.0	99 individuals with MDD + SA	119 individuals with mood disorders 39 HC	P: (M: 39, F: 60) PC: (M: 43, F: 76) HC: (M: 13, F: 26)	Plasma	A predictive association between titers of anti-*T. gondii* antibodies and history of suicide attempts (OR = 1.55; CI 1.14–2.12, *p* = 0.006)
Sugden, Moffitt [100]	USA, 2016	Prospective cohort study	Self-reported suicide attempts: Behaviors was only considered a suicide attempt if it was accompanied by a self-reported intention to die.	Range 3–38	67 individuals with SA	770 individuals without SA	M: 423, F: 414	Plasma	Suicide attempts were marginally more frequent among individuals with *T. gondii* seropositivity (OR = 2.63, 95% CI 0.97–7.14, *p* = 0.06).
Samojlowicz, Borowska-Solonynko [101]	Poland, 2013	Case–control	People who died as a result of suicide based on medico-legal autopsies.	P: median = 40 PC: median = 40 HC: median = 51	41 suicide victims	42 traffic accident victims 86 HC	P: (M: 36, F: 5) TA: (M: 39, F: 3) HC: (M: 79, F: 7)	Post mortem Plasma	With respect to the prevalence of *T. gondii* infection, no statistically significant differences were found between the study and control group (*p* = 0.09). A statistically significant result was recorded in the 38–58 age group between suicide and control groups (*p* < 0.05).
Coccaro, Lee [102]	USA, 2016	Cross-sectional	Behaviors was only considered a suicide attempt if it was accompanied by a self-reported intention to die.	P: 36.1 ± 8.3 PC: 33.7 ± 8.1 HC: 31.3 ±8.7	110 individuals with intermittent explosive disorder	110 HC 138 psychiatric controls	P: (M: 70, F: 40) PC: (M: 81, F: 57) HC: (M: 64, F: 46)	Plasma	*T. gondii* seropositive status did not predict history of suicide attempt (beta = −0.26 ± 0.46, Wald X^2^= 0.31, *p* = 0.577)
Samojlowicz, Borowska-Solonynko [103]	Poland, 2017	Case–control	Individuals who committed suicide based on medico-legal autopsies.	RB: median = 40 IRB: median = 50 HC: median = 56	126 indviduals with high-risk behavior, who committed suicide	165 HC 96 individuals with inconclusively high-risk behavior; 51 individuals with risky behavior	RB: (M: 251, F: 26) IRB: (M: 86, F: 10) HC: (M: 140, F: 25)	Post mortem plasma	A strong correlation between latent *T. gondii* infection and suicide (X^2^ = 7.04, df = 1, *p* = 0.008). A strong positive association between *T. gondii* seropositivity and suicide under the influence of alcohol (*p* = 0.003, z = 2.95, q = 0.03).
Fond, Boyer [104]	France, 2018	Cohort	Columbia Suicide Severity Rating Scale	32.0 ± 8.6	250 individuals with schizophrenia		P: (M: 184, F: 66)	Plasma	No significant association of latent Toxoplasma infection with suicide behavior was found in the models (*p* > 0.05)
Burgdorf, Trabjerg [105]	Denmark, 2019	Case–control	First episode of deliberate self-violence was defined according to the International Classification of Diseases—8th to 10th revisions. Suicide cases were identified based on medico-legal autopsies.	37.4 (no SD reported)	655 individuals with SA or suicide	2591 psychiatric controls 2724 traffic accident victims	P: (M: 278, F: 377) PC: (M: 1277, F: 1324) HC: (M: 1491, F: 1233)	Plasma	*T. gondii* infection was not statistically significantly associated with attempting or committing suicide (OR, 1.31; 95% CI, 1.10–1.56)

Abbreviations: SB, suicidal behavior; SA, suicide attempt; P, patients; PC, patient controls; HC, healthy controls; MDD, major depressive disorder; BD, bipolar disorder; TA, traffic accident victims; RB, risky behavior; IRB, inconclusively risky behavior; CI, confidence interval; OR, odds ratio; M, male; F, female.

**Table 2:** Individual studies used in this review reporting the association between the human microbiome and suicidal behavior.

Study	Country and Year	Design	SB Definition	Age in Years (Mean and First SD)	Number of Patients	Number of Control Participants	Gender (N)	Type of Biological Sample	Main Outcome
Javan, Finley [109]	USA, 2017	Case–control	Post mortem samples from the Alabama Department of Forensic Sciences in Montgomery, AL and The Office of the District One Medical Examiner in Pensacola, FL, USA.	P: 43 ± 21.4 HC: 42.8 ± 15.2	5 suicide victims	40 corpses	P: (M: 4, F: 1) HC: (M: 24, F: 16)	Post mortem liver and spleen tissues	Statistically significant difference in Chao1 richness between two 16S rRNA gene regions. In comparisons of gender and manner of death (accident, homicide, natural, suicide, or undetermined), statistically significant differences were observed (ANOVA; *p* < 0.001).
Lutz, Vangelatos [110]	Italy, 2020	Case–control	Suicide based on medico-legal routine autopsy.	Range 15–90 years.	40 suicide victims		M: 26, F: 14	Microbiota of organ tissues including brain, heart, liver, spleen, prostate, and uterus (post mortem)	Death by suicide showed a strong negative association with amplicon sequence variants (ASVs) in the family Peptostreptococcaceae (>20 log2 fold change relative to other causes of death)
Zhang, Pechal [113]	USA, 2019	Cross-sectional	Suicide based on medico-legal routine autopsy.	43.9, ranged from 18–88 years.	23 suicide cases	265 death by accident, homicide, or natural causes.	M: 105, F: 83	Post mortem sample: by swabbing the external auditory canal, eyes, nose, mouth, umbilicus, and rectum with DNA-free sterile cotton-tipped applicators	Suicide cases had significantly higher *Actinomyces* sp. counts than homicides or natural or accidental deaths (*p* < 0.01)
Ohlsson, Gustafsson [112]	USA, 2019	Cross-sectional	SB was assessed by means of the Suicide Assessment Scale (SUAS).	P: 38.5 ± 14.5 PC: 34.5 ± 11.5 HC: 34.4 ± 11.4	54 individuals with depressive symptomes + SA	13 MDD 17 HC	P: (M: 24, F:3 0) P: (M: 6, F: 7) HC: (M: 9, F: 8)	Blood plasma	The rSA group had significantly higher I-FABP and lower zonulin levels compared to both HCs and the nsMDD group (all *p* < 0.001). SUAS scores correlated significantly and positively with I-FABP and negatively with zonulin (r = −0.51, *p* < 0.001).
Thompson, Fowler [114]	USA, 2021	Cohort	Columbia Suicide Severity Rating Scale.	37.0 ± 13.8	100 psychiatric inpatients		M: 53, F: 47	Stool	No significant relationship between gut microbiota variance and SI and suicide-related behaviors in a cohort of individuals with mental disorder (*p* > 0.05).
Maes, Vasupanrajit [111]	Thailand, 2023	Cross-sectional	Columbia Suicide Severity Rating Scale.	Range: 19–58 years.	32 individuals with MDD	37 healthy controls	-	Stool	The enterotype dysbiosis index of MDD based on microbiota phyla, genera, and species was associated with the recurrence of MDD and suicidal behavior (r = 0.471, *p* < 0.001).

Abbreviations: SB, suicidal behavior; SA, suicide attempt; rSA, recent suicide attempts; P, patients; PC, patient controls; HC, healthy controls; MDD, major depressive disorder; nsMDD, individuals with major depressive disorder without suicide attempt; TA, traffic accident victims; M, male; F, female; I-FABP, intestinal fatty acid binding protein; SUAS, Suicide Assessment Scale.

## Data Availability

No new data were created or analyzed in this study. Data sharing is not applicable to this article.

## Supplementary Materials

The following supporting information can be downloaded at: https://www.mdpi.com/article/10.3390/jcm13020593/s1, PRISMA checklist [136].
